# Associations Between Smartphone Addiction and Physical Education Learning Engagement Among Chinese University Students: A Variable-Centered and Person-Centered Analysis

**DOI:** 10.3390/bs16091683

**Published:** 2026-09-18

**Authors:** Shiyu Zhang, Huawei Chen, Liang Li

**Affiliations:** 1 Physical Education College, Jiangxi Normal University, Nanchang 330022, China; zhangshiyu@jxnu.edu.cn; 2 Department of Physical Education, Nanjing University of Aeronautics and Astronautics, Nanjing 210016, China

**Keywords:** smartphone addiction, physical education learning engagement, self-esteem, physical activity participation, university students

## Abstract

Smartphone addiction is increasingly prevalent among university students, yet its association with physical education learning engagement remains underexplored. This study investigated the relationship between smartphone addiction and physical education learning engagement using variable-centered and person-centered approaches, and further examined the serial indirect association involving self-esteem and physical activity participation. A cross-sectional survey was conducted among 2118 university students in Jiangxi Province, China. Participants completed measures of smartphone addiction, self-esteem, physical activity participation, and physical education learning engagement. Pearson correlations, multiple regression analyses, and analyses of indirect associations with bias-corrected bootstrapping were performed in SPSS, and latent profile analysis was conducted in Mplus. Smartphone addiction was negatively associated with self-esteem (*β* = −0.446, *p* < 0.001), physical activity participation (*β* = −0.231, *p* < 0.001), and physical education learning engagement (*β* = −0.152, *p* < 0.001). Self-esteem and physical activity participation showed significant indirect associations in the relationship between smartphone addiction and physical education learning engagement, including a significant serial indirect association through both variables. Person-centered analyses identified three smartphone addiction profiles, namely a low-smartphone-addiction group, moderate-smartphone-addiction group, and high-smartphone-addiction group, accounting for 22.10%, 44.24%, and 33.66% of the sample, respectively. Significant gradient differences were observed across these profiles in self-esteem, physical activity participation, and physical education learning engagement. Smartphone addiction was negatively associated with physical education learning engagement among university students, both directly and indirectly through self-esteem and physical activity participation. Distinct smartphone addiction profiles were also identified, with corresponding differences in psychological and behavioral characteristics. Given the cross-sectional and self-report design, the findings should be interpreted as correlational rather than causal.

## 1. Introduction

With the rapid expansion of digital technology, smartphones have become deeply integrated into university students’ learning, communication, and daily routines. Although smartphones provide important benefits for information access, social connection, and online learning, excessive and poorly regulated use may develop into smartphone addiction (SA) (Nikolic et al., 2023). Among university students, SA has been associated with anxiety, sleep problems, impaired attention, poorer self-regulation, and reduced well-being (Demirci et al., 2015; Elhai et al., 2016; Li et al., 2020; Rathakrishnan et al., 2021). In a study of Chinese college students, the proportion screening positive for SA reached 36.6%, although such estimates vary across studies depending on the sample and assessment criteria used (Mei et al., 2023). Given the widespread use of smartphones in higher education, clarifying how SA relates to students’ learning has become an important research issue.

Most previous research on SA has focused on mental health, academic performance, and general learning engagement (Meng et al., 2025; Rathakrishnan et al., 2021), whereas less attention has been paid to physical education learning engagement (PELE). PELE refers to students’ sustained behavioral, emotional, and cognitive involvement in physical education learning activities (Li & Saibon, 2025; Stringfellow et al., 2024). Physical education typically involves visible bodily participation, movement practice, immediate performance feedback, physical challenge, and peer observation (Sasaki, 2015; T. Zhang & Zhang, 2017). Accordingly, PELE depends not only on general academic motivation but also on willingness to participate physically, persistence in effortful activity, and comfort in socially evaluative contexts. For university students, physical education is not merely a curricular component; it is also an important setting for motor skill development, stress relief, health promotion, and the cultivation of lifelong physical activity habits (Eime et al., 2013; Hastie et al., 2022; Zhou et al., 2025). Findings concerning general academic engagement therefore cannot be assumed to generalize directly to PE settings. Examining the association between SA and PELE may thus extend current understanding of how problematic smartphone use relates to learning in a context that is both educationally and health relevant.

University students are a particularly relevant population for examining these associations. Emerging adulthood is characterized by increasing autonomy, reduced external monitoring, heightened academic and social demands, and ongoing identity development, all of which may shape smartphone use and self-regulation (Coyne et al., 2019). In the context of Chinese higher education, physical education also remains an important institutional setting for organized physical activity and health-related learning. For many students, university may be one of the last structured educational stages in which physical education can systematically influence participation habits, engagement, and health-related development. Understanding how SA relates to PELE in this population may therefore have both educational and public health significance.

The present study was informed by Self-Determination Theory and cognitive–behavioral models of problematic technology use (Ryan & Deci, 2000). Cognitive–behavioral models provide the primary theoretical basis for understanding how smartphone addiction (SA) may influence individuals’ psychological functioning. According to these models, prolonged problematic smartphone use may create maladaptive cognitive and behavioral cycles characterized by attentional fragmentation, a preference for immediate gratification, maladaptive coping strategies, and excessive online social comparison, while also contributing to repeated failures of self-regulation. Over time, these tendencies may reduce individuals’ willingness and capacity to invest effort in demanding offline activities (Elhai et al., 2016; Rathakrishnan et al., 2021; Wang et al., 2025). Self-Determination Theory further explains learning and behavioral engagement from a motivational perspective, proposing that active participation and sustained engagement depend on the satisfaction of basic psychological needs, particularly competence and autonomy (Gómez-López et al., 2025). Taken together, these two perspectives suggest that SA, as a risk factor reflecting deficits in self-regulation, may not only directly undermine students’ concentration and learning states, but also indirectly influence physical education learning engagement through both psychological and behavioral pathways. At the same time, alternative or reciprocal relationships are also theoretically plausible; for example, students with lower self-esteem may be more likely to engage in compensatory smartphone use. Consistent with these perspectives, previous studies have shown that SA is associated with lower learning engagement, poorer academic self-efficacy, and lower physical activity (Hsieh, 2025; Meng et al., 2025). However, direct evidence linking SA to PELE remains limited, and the mechanisms underlying this relationship have not been sufficiently examined.

Among the potential mechanisms, self-esteem (SE) may be particularly relevant. SE reflects an individual’s overall evaluation of self-worth and competence and is an important psychological resource for persistence, coping, and adaptive functioning in learning contexts (Dou et al., 2024; Orth & Robins, 2014). Students with higher SE are generally more likely to maintain confidence, tolerate challenge, and remain positively engaged in learning, whereas lower SE is often associated with self-doubt, avoidance, and motivational deficits (Acosta-Gonzaga, 2023; Dadandı & Yazıcı, 2024; Liao et al., 2025). Prior research has consistently reported a negative association between problematic smartphone use and SE (Hawi & Samaha, 2017; Ko et al., 2025), possibly because excessive smartphone dependence may coincide with heightened social comparison, reduced offline interaction, and repeated experiences of self-regulation failure (Bottaro & Faraci, 2025; C. Chen et al., 2023; Servidio et al., 2021). SE has also been linked to stronger engagement and better psychological adjustment in educational contexts (Acosta-Gonzaga, 2023; Dadandı & Yazıcı, 2024; Orth & Robins, 2022). On this basis, SE may help explain the negative association between SA and PELE.

Physical activity participation (PAP) may represent another relevant pathway. PAP refers to individuals’ actual participation in exercise or sport-related activities, such as the frequency and continuity of their involvement (Ross et al., 2016). Although PAP is closely related to PELE, the two constructs are not equivalent. PAP concerns the extent to which students engage in physical activity across relevant contexts, whereas PELE refers specifically to the quality of their behavioral, emotional, and cognitive engagement in physical education learning settings (Li & Saibon, 2025; X. Liu & Wang, 2026). Thus, PAP reflects broader behavioral participation, whereas PELE captures context-specific educational engagement; some overlap with the behavioral dimension of PELE is possible, but the constructs differ in scope and psychological content. Earlier findings indicate that SA is associated with more sedentary behavior, lower physical activity, and weaker exercise motivation (Nambirajan et al., 2025; Ye et al., 2025). Excessive smartphone use may therefore reduce students’ willingness or persistence in PAP, which may in turn relate to lower PELE (Lizandra et al., 2019; Xu et al., 2026).

In addition to their independent indirect roles, SE and PAP may also be linked in a serial manner within the hypothesized model. As a psychological resource, SE may shape whether students are willing to enter and persist in activity contexts involving bodily display, challenge, and social evaluation. Higher SE may support exercise confidence, physical self-identity, and proactive participation, whereas lower SE may be associated with avoidance of physically demanding or publicly visible activities (Chung et al., 2021; Guo et al., 2025; Moffitt, 2024). In this way, lower SE may be associated with lower PAP, which may then relate to weaker PELE. At the same time, alternative directional relationships are theoretically plausible. For example, regular physical activity may contribute to higher SE, and stronger engagement in physical education may also encourage broader physical activity participation.

**Figure 1:**
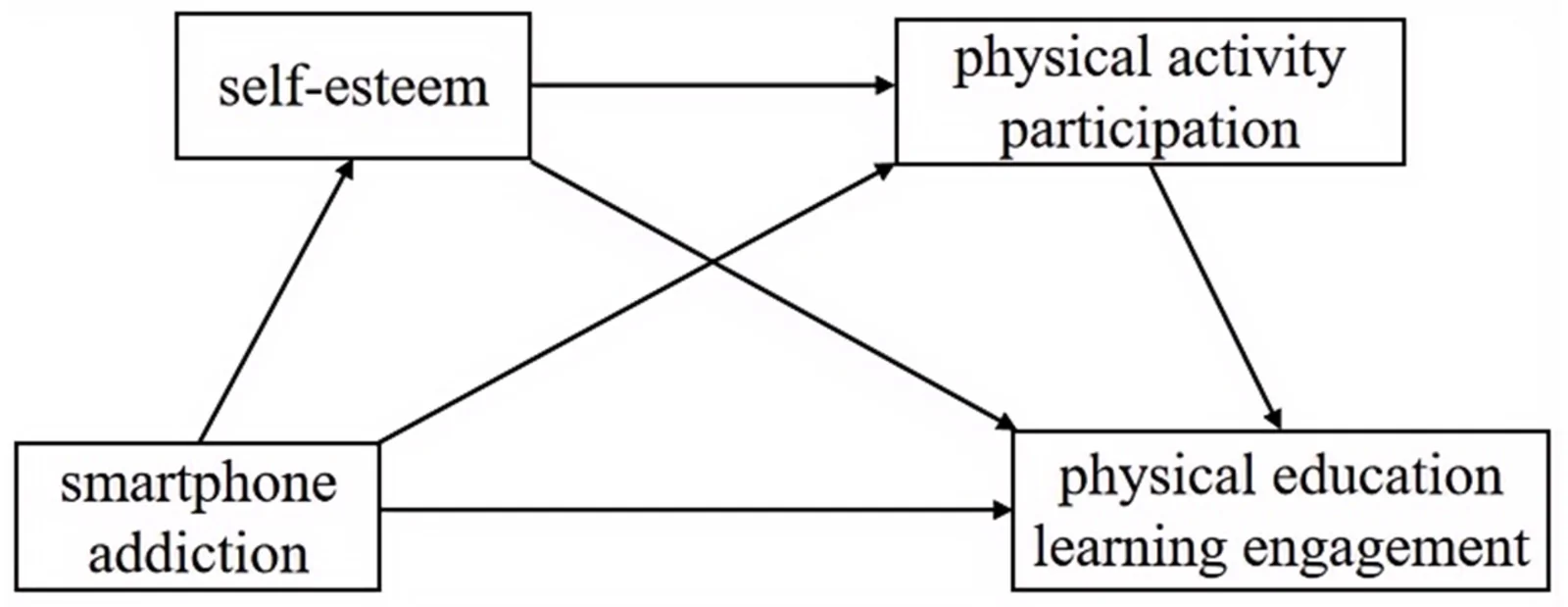
The hypothesized model diagram.

Another limitation in the existing literature is the predominant reliance on variable-centered approaches, which estimate average associations but provide limited insight into heterogeneity in SA. A person-centered approach may therefore be informative because university students may differ not only in overall severity of SA but also, potentially, in the relative configuration of its dimensions. Latent profile analysis can help identify empirically derived subgroups and clarify whether PELE differs systematically across them. Even if the resulting profiles mainly reflect severity differences rather than qualitatively distinct patterns, such findings remain meaningful because they identify risk strata that may be relevant for targeted intervention (L. Chen et al., 2025; J. Zhang & Wang, 2024). In this respect, the profile analysis was exploratory with regard to the number and exact characteristics of the latent profiles (Figure 1).

Accordingly, the present study combined variable-centered and person-centered approaches to examine the association between SA and PELE among Chinese university students. Accordingly, the following hypotheses were proposed:

**Hypothesis 1.**  *SA is negatively associated with university students’ PELE.*

**Hypothesis 2.**  *The association between SA and PELE is indirect through SE.*

**Hypothesis 3.**  *The association between SA and PELE is indirect through PAP.*

**Hypothesis 4.**  *The association between SA and PELE is serially indirect through SE and PAP.*

## 2. Materials and Methods

### 2.1. Participants

An a priori power analysis was conducted using G*Power 3.1 based on an F test for linear multiple regression (fixed model, *R*^2^ deviation from zero). A medium effect size (*f*^2^ = 0.15), a significance level of α = 0.05, statistical power of 1 − *β* = 0.90, and a maximum of five predictors were specified (Kang, 2021). The analysis indicated a minimum sample size of 119 participants. Additionally, a participant-to-item ratio of 10–15:1 was applied in accordance with methodological recommendations in psychological and behavioral research (Lund, 2021). Based on 47 total questionnaire items, the estimated appropriate sample size ranged from 470 to 705 participants (Lakens, 2022). The final sample size of 2118 exceeded both criteria.

Participants were drawn from three public universities in Jiangxi Province, China, through convenience sampling. The participating institutions included a comprehensive university, a normal university, and a science- and technology-oriented university, thus representing different institutional types within the higher education system. Although this sampling method may increase the risk of selection bias and limit the generalizability of the findings, it was considered appropriate for the present study because the research was exploratory in nature and subject to practical constraints in participant recruitment. To enhance sample diversity, students from a broad range of academic disciplines were invited to participate, and both male and female students were included, resulting in a relatively heterogeneous sample of university students.

The study targeted registered full-time undergraduate students at the participating universities who were between 18 and 23 years of age and were expected to have current or recent experience with physical education (PE) classes, as PE courses are typically mandatory in the first two years of study at the participating institutions, thereby providing an appropriate reference point for the PE engagement items. Data were collected between 8 April 2026, and 13 May 2026. The survey was administered online and distributed through student organizations and social media platforms, such as WeChat and QQ. Participation was voluntary, and written informed consent was obtained from all participants before the survey was completed. This study was approved by the Institutional Review Board of the Physical Education College, Jiangxi Normal University (protocol code: No. IRB-JXNU-PEC-2026010).

A total of 2368 questionnaires were distributed. Responses were screened sequentially according to predefined validity criteria to avoid double counting. First, questionnaires with a completion rate below 75% were removed (n = 34). Accordingly, questionnaires completed in an unreasonably short time were excluded (n = 76), questionnaires with invariant responses were removed (n = 62), and participants who failed the attention-check items were excluded (n = 78). After data screening, 2118 valid responses were retained for analysis. Participants were aged 18 to 23 years, with a mean age of 19.27 years (SD = 1.08). Among them, male students accounted for 47.92% of the sample, and female students accounted for 52.08%. In terms of year of study, 694 (32.77%) were first-year, 1263 (59.63%) second-year, 144 (6.80%) third-year, and 17 (0.80%) fourth-year students.

### 2.2. Measurement

#### 2.2.1. Utrecht Work Engagement Scale for Students (UWES-S)

PELE was assessed using the UWES-S (Schaufeli et al., 2002), which was translated and validated by Fang et al. (2008). To adapt the scale to the physical education context, the instruction was modified to refer specifically to “physical education classes” (e.g., “During physical education classes, I feel energetic and motivated.”), while all item wordings remained unchanged. Although initially validated in middle school students (Miao et al., 2024), we applied this version to university students for the following reasons: (a) the UWES-S measures domain-specific engagement rather than age-specific engagement; (b) the wording changes pertain to the physical education context, which is equally relevant across educational levels; and (c) previous studies have successfully used this version in university samples. The scale comprises 17 items rated on a 5-point Likert scale, with items summed to generate a total score. Higher scores indicate greater PELE. In this study, Cronbach’s alpha for the total scale was 0.916. To further validate the PE-adapted UWES-S in the present sample, a confirmatory factor analysis (CFA) with robust maximum likelihood (MLR) estimation was conducted to test its unidimensional structure. The model demonstrated acceptable fit: *χ*^2^(119) = 724.10, *p* < 0.001, CFI = 0.923, TLI = 0.912, RMSEA = 0.049, 90% CI [0.046, 0.052], SRMR = 0.039. All standardized factor loadings were statistically significant and ranged from 0.581 to 0.675, supporting the unidimensionality of the scale in the present sample.

#### 2.2.2. Mobile Phone Addiction Index (MPAI)

SA was assessed using the MPAI (Leung, 2008). The scale consists of 17 items covering four dimensions: inability to control craving, anxiety and withdrawal, productivity loss, and escapism. A sample item is “I feel uneasy without my mobile phone.” All items were rated on a 5-point Likert scale, with higher scores indicating higher levels of SA. Cronbach’s alpha for the total scale in this study was 0.942.

#### 2.2.3. Physical Activity Rating Scale-3 (PARS-3)

PAP was assessed using the PARS-3, revised by Liang (1994). The scale consists of three items assessing exercise intensity, duration, and frequency (e.g., “What is the intensity of my physical exercise?”). Each item is rated on a 5-point scale ranging from 1 to 5. The total physical activity score is calculated as intensity × (duration − 1) × frequency, yielding a possible range from 0 to 100, with higher scores indicating greater PAP. The subtraction of 1 from the duration score ensures that the lowest duration category is scored as 0. Previous validation studies have reported a test–retest reliability of 0.82 for the Chinese version of the PARS-3 (Liang, 1994), indicating acceptable temporal stability.

#### 2.2.4. Self-Esteem Scale (SES)

SE was assessed using the SES (Alessandri et al., 2015). The scale includes 10 items, with five positively worded items and five negatively worded items (e.g., “I take a positive attitude toward myself.”). Item scores were summed to generate a total score, with higher scores indicating greater SE. Cronbach’s alpha for the total scale in this study was 0.791.

### 2.3. Statistical Analysis

All analyses were conducted using SPSS 26.0 and Mplus 8.3. Preliminary analyses included descriptive statistics, Pearson correlation analysis, and tests of common method bias. Multiple regression analyses were used to examine the associations among SA, SE, PAP, and PELE while controlling for gender and age. A serial indirect association analysis was conducted using Model 6 of the PROCESS macro (Hayes et al., 2017), with gender and age included as covariates. The significance of the indirect effects was evaluated using a bias-corrected bootstrap approach with 5000 resamples; effects were considered significant when the 95% confidence interval excluded zero (Cheung & Lau, 2008).

LPA was conducted in Mplus 8.3 using a person-centered approach. The 17 individual items of the MPAI were entered directly as continuous profile indicators, rather than using the four subscale scores, because item-level indicators preserve greater heterogeneity in response patterns and may better capture nuanced profile differences. Missing data were handled using full-information maximum-likelihood estimation, and the robust maximum-likelihood estimator (MLR) was applied. Indicator means were freely estimated in each profile. In line with the default Mplus specification for mixture models, indicator variances were constrained to be equal across profiles, and within-profile covariances were constrained to zero (local independence). To reduce the risk of local solutions and facilitate model convergence, we specified 200 random starting values, 50 final-stage optimizations, and 20 initial-stage iterations, using four processors for parallel computation. Model convergence was evaluated by confirming that the best log-likelihood value was replicated. Model estimation began with a one-profile solution and proceeded by successively increasing the number of latent profiles. Model fit was evaluated using the Akaike Information Criterion (AIC), Bayesian Information Criterion (BIC), adjusted Bayesian Information Criterion (aBIC), entropy, the Lo–Mendell–Rubin adjusted likelihood ratio test (LMR-LRT), and the bootstrap likelihood ratio test (BLRT). Lower AIC, BIC, and aBIC values indicate better model fit, whereas higher entropy indicates better classification accuracy (Huang et al., 2025). The final model was selected based on statistical fit, class size, substantive interpretability, and parsimony. After identifying the optimal latent profile solution, differences in SE, PAP, and PELE across the latent profiles were examined using the Bolck–Croon–Hagenaars (BCH) procedure. The resulting profiles were named according to their indicator characteristics and substantive interpretability.

## 3. Results

### 3.1. Measurement Model, Common Method Bias, Normality, and Multicollinearity Assessment

A confirmatory factor analysis (CFA) was conducted to examine the measurement structure of SA, SE, and PELE. The three-factor model showed acceptable fit: *χ*^2^(874) = 4225.60, *p* < 0.001, CFI = 0.946, TLI = 0.928, RMSEA = 0.041, 90% CI [0.040, 0.042], and SRMR = 0.035. All standardized factor loadings exceeded 0.50, supporting the adequacy of the measurement model. Because the subsequent mediation analyses were conducted using the total scores of SA, SE, and PELE, internal consistency reliability for these constructs was evaluated using Cronbach’s alpha, as reported in the Section 2.2. PAP was treated as an observed composite variable based on the scoring procedure of the PARS-3 and was therefore not modeled as a reflective latent construct in the CFA.

Before formal analysis, common method variance was assessed using Harman’s single-factor test (Podsakoff et al., 2003). An unrotated exploratory factor analysis of all measurement items extracted eight factors with eigenvalues greater than 1, and the first factor explained 27.09% of the total variance, which was below the commonly used threshold of 40%. In addition, a single-factor confirmatory factor analysis in Mplus showed poor model fit (CFI = 0.565, TLI = 0.540, RMSEA = 0.116, SRMR = 0.107) (Podsakoff et al., 2024). Although the results did not indicate a severe common method bias problem, common method bias cannot be excluded given the cross-sectional self-report design. Subsequently, the distributional properties of the data were assessed. As shown in Table 1, all variables exhibited acceptable distributional properties. The absolute values of skewness were below 3, and the absolute values of kurtosis were below 10, indicating no serious departure from normality (Markus, 2012). Variance inflation factors (VIFs) were further calculated to assess multicollinearity among the study variables. The VIF values ranged from 1.327 to 1.413, with a maximum value of 1.413, which was well below the commonly accepted cutoff of 5. Therefore, multicollinearity was not considered a serious concern in the present study.

### 3.2. Correlation Analysis

As shown in Table 2, SA was significantly negatively correlated with SE (*r* = −0.447, *p* < 0.01), PAP (*r* = −0.396, *p* < 0.01), and PELE (*r* = −0.353, *p* < 0.01). In contrast, SE was significantly positively correlated with PAP (*r* = 0.455, *p* < 0.01) and PELE (*r* = 0.405, *p* < 0.01), and PAP was significantly positively correlated with PELE (*r* = 0.415, *p* < 0.01). All correlations were statistically significant.

### 3.3. Regression Analysis

As shown in Table 3, all multiple regression models were statistically significant. In the model with SE as the outcome, the overall model explained 20.1% of the variance in SE (*R*^2^ = 0.201, *F* = 176.749, *p* < 0.001). SA showed a moderate negative association with SE (*β* = −0.446, *p* < 0.001), whereas gender (*β* = 0.016, *p* = 0.402) and age (*β* = 0.006, *p* = 0.762) were not significantly associated with SE.

In the model with PAP as the outcome, the regression model explained 28.3% of the variance (*R*^2^ = 0.283, *F* = 208.631, *p* < 0.001). SE (*β* = 0.344, *p* < 0.001) and SA (*β* = −0.231, *p* < 0.001) showed comparatively larger associations with PAP, whereas the effects of gender (*β* = 0.164, *p* < 0.001) and especially age (*β* = 0.042, *p* = 0.022) were relatively small despite reaching statistical significance.

**Figure 2:**
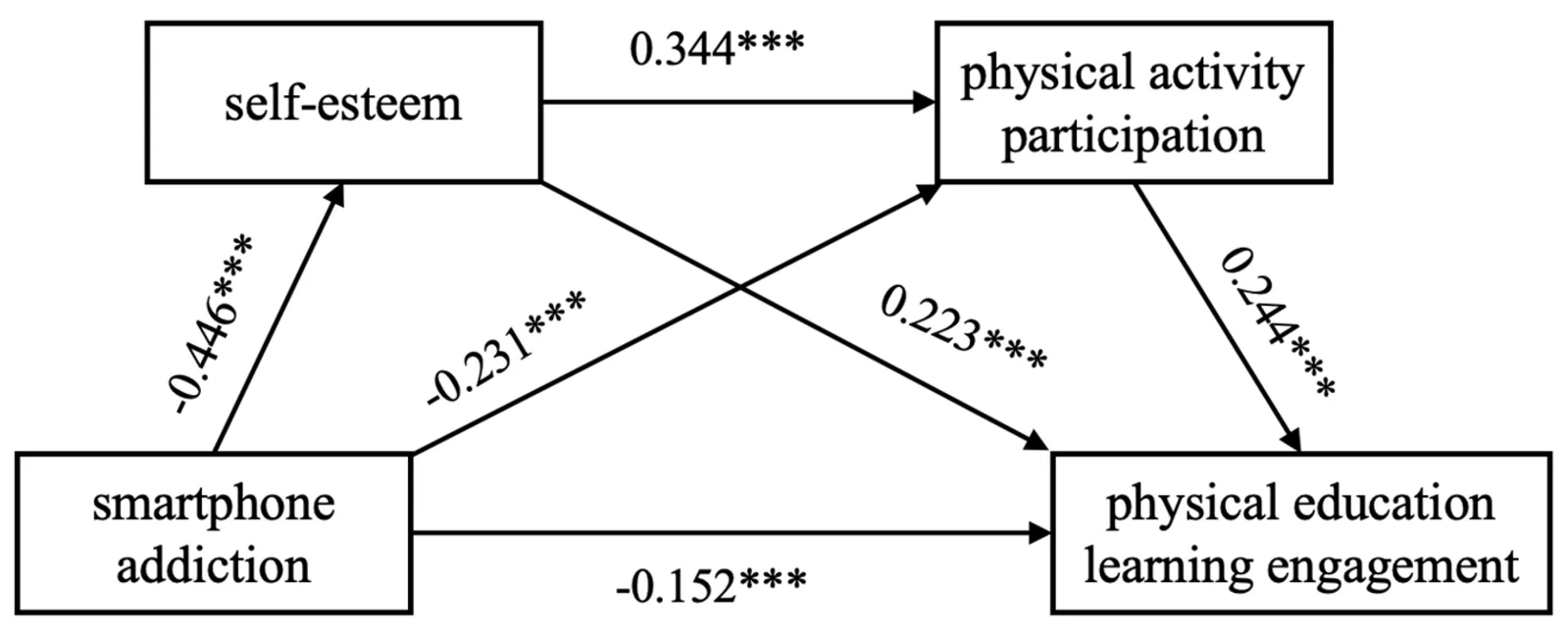
Serial indirect association model of SA and PELE. Note. *** *p* < 0.001.

In the model with PELE as the outcome, the regression model was also statistically significant and explained 25.3% of the variance (*R*^2^ = 0.253, *F* = 129.013, *p* < 0.001). PAP (*β* = 0.244, *p* < 0.001) and SE (*β* = 0.223, *p* < 0.001) were the strongest positive correlates of PELE, while SA was negatively associated with PELE (*β* = −0.152, *p* < 0.001). By contrast, the associations of gender (*β* = 0.053, *p* = 0.006) and age (*β* = −0.038, *p* = 0.047) with PELE were statistically significant but small in magnitude. Overall, the findings suggest that SA was negatively associated with SE, PAP, and PELE, whereas SE and PAP were positively associated with PELE (see Figure 2). However, the effects of age and gender were generally small and should be interpreted with caution in terms of practical significance.

### 3.4. Indirect Association Analysis

As shown in Table 4, the total association between SA and PELE was significant (*β* = −0.346), and the direct association remained significant after accounting for the mediators (*β* = −0.152). The total indirect association was significant (*β* = −0.194, BootSE = 0.013, 95% CI [−0.221, −0.167]). Specifically, the indirect association through SE was significant (*β* = −0.100, BootSE = 0.012, 95% CI [−0.122, −0.076]), accounting for 51.40% of the total indirect association. The indirect association through PAP was also significant (*β* = −0.057, BootSE = 0.007, 95% CI [−0.072, −0.043]), accounting for 29.05%. In addition, the serial indirect association through SE and PAP was significant (*β* = −0.038, BootSE = 0.004, 95% CI [−0.046, −0.029]), accounting for 19.55%. Pairwise comparisons showed significant differences among the three indirect associations, as all bootstrap confidence intervals for the contrasts excluded zero.

### 3.5. Latent Profile Analysis of Smartphone Addiction

As shown in Table 5, the AIC, BIC, and adjusted BIC values decreased as additional profiles were extracted, indicating incremental improvement in statistical fit. However, the magnitude of improvement was greatest when moving from the 2-profile to the 3-profile solution, whereas subsequent reductions from three to 4 profiles and from four to 5 profiles became progressively smaller, suggesting diminishing incremental gains in fit. Therefore, the three-, four-, and five-profile solutions were compared carefully rather than selecting the final model solely on the basis of information criteria. The three-profile solution showed good classification accuracy (entropy = 0.922), significant likelihood-ratio test results, and adequate class proportions. Although the four- and five-profile solutions yielded lower information criteria, visual inspection of the profile plots suggested that the additional profiles primarily reflected further severity-based subdivisions rather than qualitatively distinct subgroups. The five-profile solution retained a significant BLRT result, but the VLMR test was no longer statistically significant at the conventional level (*p* = 0.0518). Moreover, in large samples, the BLRT may be sensitive to the addition of profiles and may continue to support more complex solutions even when the added classes offer limited substantive interpretive value. Across the three-, four-, and five-profile solutions, the item-response curves were broadly parallel, indicating that the latent profiles differed mainly in overall symptom level rather than in qualitatively distinct response configurations. Accordingly, considering statistical fit, class proportions, classification accuracy, substantive interpretability, and parsimony together, the three-profile solution was retained as the most appropriate model. The retained profiles should therefore be understood primarily as severity-based subgroups rather than qualitatively distinct forms of smartphone addiction. To facilitate independent evaluation of this decision, the profile plots for the 4-profile and 5-profile solutions are provided in the Supplementary Materials.

**Figure 3:**
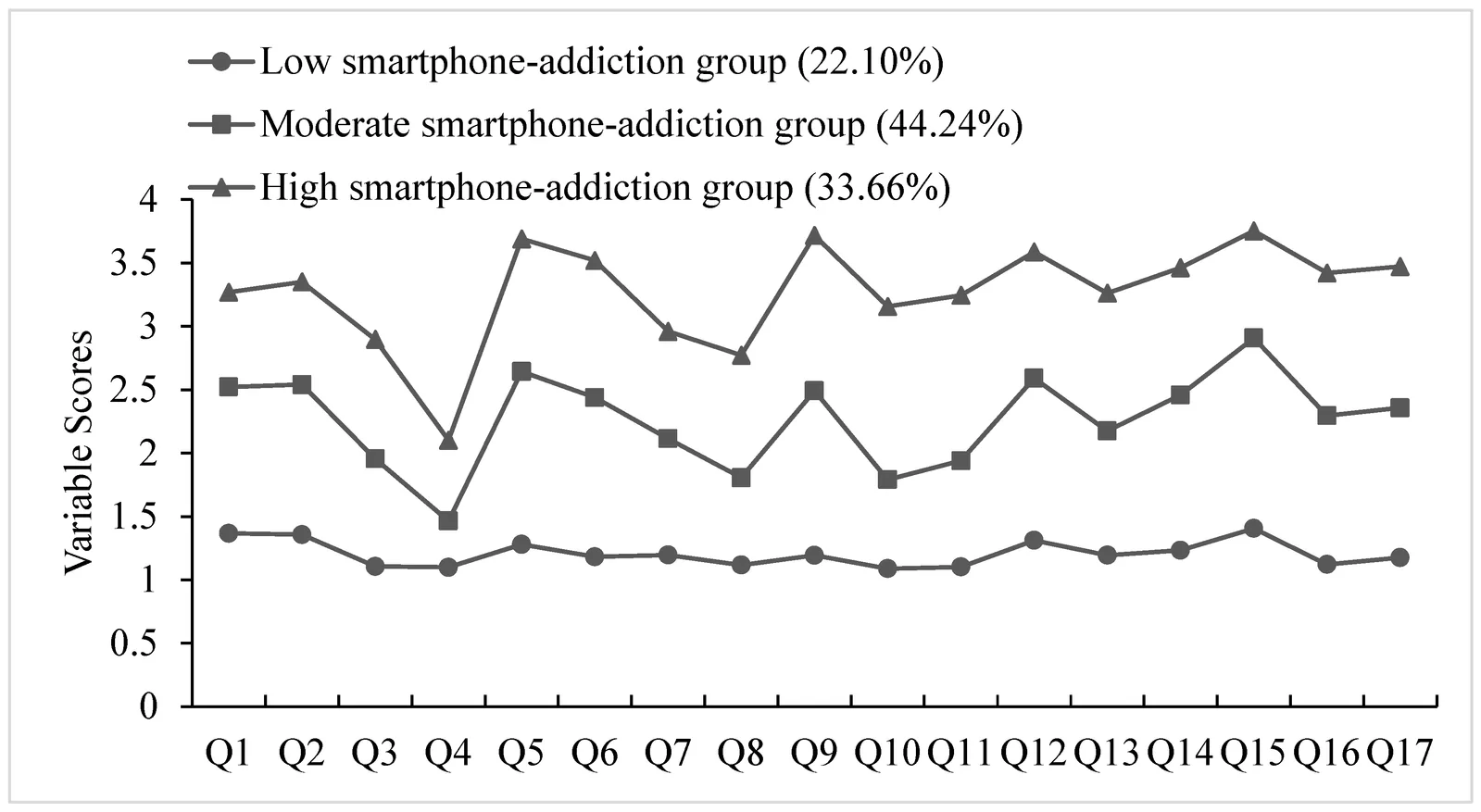
Latent profile of smartphone addiction.

As depicted in Figure 3, the three latent profiles showed broadly similar patterns across the 17 smartphone-addiction items but differed mainly in their overall level of item endorsement, suggesting severity-based rather than qualitatively distinct heterogeneity. Specifically, Class 1 was characterized by consistently low scores across items and was therefore labeled the low-smartphone-addiction group (22.10%). Class 2 showed moderate levels across items and was labeled the moderate-smartphone-addiction group (44.24%). Class 3 showed consistently high scores across items and was labeled the high-smartphone-addiction group (33.66%).

### 3.6. Differences in SE, PAP, and PELE Across Latent Profiles

As shown in Table 6, BCH analyses revealed significant differences in SE, PAP, and PELE across the three latent profiles (all *p* < 0.001). For all three distal outcomes, the low-smartphone-addiction group (C1) showed the highest estimated means, followed by the moderate-smartphone-addiction group (C2) and the high-smartphone-addiction group (C3). Specifically, SE differed significantly across profiles, *χ*^2^(2) = 478.542, *p* < 0.001, with estimated means of 27.481, 22.672, and 21.441 for C1, C2, and C3, respectively. PAP also differed significantly, *χ*^2^(2) = 379.793, *p* < 0.001, with estimated means of 60.688, 38.107, and 30.297, respectively. Similarly, PELE differed significantly across profiles, *χ*^2^(2) = 245.577, *p* < 0.001, with estimated means of 64.372, 55.780, and 52.665, respectively. Pairwise comparisons showed that all class differences were significant for all three outcomes (all *p* < 0.001). Descriptive effect sizes for pairwise group differences, calculated based on most-likely class assignment, are presented in Table 7.

## 4. Discussion

### 4.1. Association Between Smartphone Addiction and Physical Education Learning Engagement

In the present study, higher SA was associated with lower PELE. This result is broadly consistent with previous studies linking problematic smartphone use to poorer academic engagement and less favorable learning-related outcomes (Hsieh, 2025; Meng et al., 2025; Sunday et al., 2021). According to potential explanations proposed in the previous literature, in physical education contexts, this association may be related to attentional fragmentation, reduced self-regulation, and increased behavioral displacement, all of which may be incompatible with the sustained concentration and active participation required in physical education (Levy et al., 2016; Uncapher et al., 2017; Wilmer et al., 2017). However, given the large sample size, statistical significance should be interpreted alongside effect size. According to Cohen’s benchmarks, the observed associations were generally small to moderate, and the standardized association between SA and PELE was modest. The regression models also explained limited variance. Accordingly, SA should be viewed as a relevant, but not dominant, correlate of PELE.

This finding is also theoretically plausible in light of the contextual features of physical learning. PELE is not merely reflected in class attendance or superficial participation; rather, it encompasses behavioral, emotional, and cognitive dimensions, emphasizing students’ active involvement, concentration, effort, and meaning-making during class (X. Liu & Wang, 2026; B. Zhang et al., 2020). Compared with many conventional classroom settings, physical education classes place greater demands on real-time response, physical practice, rule implementation, and peer interaction, and thus require higher levels of sustained attention, self-control, and situational involvement (Hastie et al., 2022; Stringfellow et al., 2024). These demands may make sustained attention and self-regulation especially relevant. By contrast, SA is often characterized by fragmented attention, reward-seeking, media multitasking, and sensitivity to external cues such as notifications (Levy et al., 2016; Uncapher et al., 2017; Wilmer et al., 2017; Yap & Lim, 2013). Such tendencies may be less compatible with the demands of physical learning. Recent research has also suggested that digital distraction in physical education settings is associated with lower cognitive availability and lower perceived autonomy support, which may coincide with weaker classroom engagement (Xu et al., 2026).

In addition to competition for cognitive resources, SA may also be linked to lower PELE through a time-displacement process. Previous studies have shown that problematic smartphone use is closely associated with lower levels of physical activity, greater sedentary behavior, and fewer daily steps (Lin et al., 2022; Tao et al., 2026; Yu et al., 2025). A systematic review and meta-analysis likewise reported a stable negative association between SA and physical activity among university students (Yu et al., 2025). This suggests that SA may represent not only a source of cognitive distraction, but also a lifestyle-related factor that directly competes with movement-related behaviors. When students devote substantial amounts of time to online entertainment, social media scrolling, or passive information consumption, they may have fewer opportunities to engage in extracurricular exercise, accumulate motor-skill experience, and develop positive sport-related experiences. Such patterns may, in turn, be associated with reduced willingness and readiness to invest in physical learning.

These findings should nevertheless be interpreted with caution. Smartphones are not inherently harmful; their implications likely depend on the content, purpose, timing, and controllability of use. Prior work suggests that the main concerns are more closely tied to uncontrolled, compensatory, and functionally impairing patterns of use than to smartphone use itself (C. Liu, 2023). Thus, the negative association observed here should not be interpreted as applying uniformly to all forms of smartphone use.

### 4.2. Indirect Association of Self-Esteem

The present study further found that SE showed a significant indirect association in the relationship between SA and PELE. This finding suggests that the negative association between SA and PELE may also be understood in relation to students’ psychological resources.

This result is consistent with previous studies reporting that problematic smartphone use is associated with low SE. Existing research indicates that SE is closely related to SA and is also associated with various psychosocial factors (Dou et al., 2024; Ko et al., 2025; Pérez-Torres, 2024; Rhodes et al., 2017). For example, Ko et al. (2025) reported that changes in SE were significantly associated with changes in smartphone dependence among Korean adolescents. Dou et al. (2024) showed that depression and anxiety may be linked to SA through SE. C. Chen et al. (2023) and P. Zhang et al. (2025) further highlighted the central role of SE in the development of SA through pathways involving social avoidance, peer relationships, and upward social comparison in online environments. At the same time, both classic and recent studies have consistently conceptualized SE as a core psychological resource for adaptive functioning, closely related to persistence, agency, learning adjustment (Orth & Robins, 2014, 2022). Students with higher SE tend to report better adjustment, greater engagement, and more favorable learning-related outcomes (Acosta-Gonzaga, 2023; Ni et al., 2025).

Several interpretations may help account for this pattern. Problematic smartphone use is often intertwined with fear of missing out, dependence on external validation, and maladaptive emotion regulation (Dou et al., 2024; Elhai et al., 2016; Servidio et al., 2021). Greater reliance on immediate digital feedback for self-worth may be associated with a more fragile and unstable self-system characterized by stronger dependence on external evaluation. In social media-saturated environments, continuous exposure to others’ idealized self-presentations may also be related to higher levels of self-doubt and comparison pressure, which may coincide with lower stable SE (Cingel et al., 2022; Hawi & Samaha, 2017; Pérez-Torres, 2024). In this sense, the relevance of SA may lie not only in excessive use, but also in the self-evaluative processes that accompany such use.

SE may be particularly relevant in physical education settings, where students often encounter bodily display, public evaluation, performance feedback, and peer comparison. Students with higher SE may feel more comfortable attempting challenging tasks, accepting correction, tolerating temporary failure, and remaining engaged. By contrast, students with lower SE may be more inclined to withdraw because of embarrassment concerns, evaluation anxiety, or low confidence (Villodres et al., 2025). SE may therefore be viewed as an important correlate of active participation and persistence in physical learning. At the same time, it is unlikely to be the only relevant factor. Previous work has also highlighted the possible roles of self-control, social anxiety, perceived stress, self-efficacy, and psychological resilience (Huang et al., 2025; Wang et al., 2025; Ye et al., 2025; T. Zhang et al., 2025). The present results therefore support the relevance of SE as one associated factor rather than a complete explanation.

### 4.3. Indirect Association of Physical Activity Participation

PAP was also statistically associated with the relationship between SA and PELE. This finding indicates that the association between SA and PELE may also be understood in relation to students’ daily movement behaviors and exercise habits.

This result is highly consistent with recent evidence. Multiple studies have shown that problematic smartphone use is significantly associated with lower levels of physical activity and greater sedentary behavior (Lizandra et al., 2019; Nambirajan et al., 2025; Tao et al., 2026). For example, Burnell et al. (2026) found a negative association between smartphone use time and step counts among adolescents. Lin et al. (2022) suggested that SA may be linked to physical activity among Chinese university students through motivational and self-efficacy-related processes. A systematic review and meta-analysis further supported this pattern among university students (Yu et al., 2025). The present study extends the existing literature by indicating that lower PAP may be one behavioral correlate of the association between SA and lower PELE.

This interpretation also fits the characteristics of physical learning. Regular physical activity is often associated with better fitness, greater movement familiarity, and stronger perceived competence, all of which may be relevant to students’ willingness to participate and invest effort in physical education. Physical activity has also been linked to improved mood, lower stress, enhanced cognitive activation, and better executive functioning (Lubans et al., 2016; Rhodes et al., 2017; Rhodes & Kates, 2015; Wilhite et al., 2023), all of which may coincide with stronger behavioral and cognitive engagement during class. In addition, habitual participation in physical activity may be associated with positive exercise experiences and greater continuity between everyday active behavior and formal physical learning settings.

Importantly, PAP and PELE are related but distinct constructs. PAP refers primarily to students’ broader participation in physical activity behavior in daily life or outside the classroom, whereas PELE refers to students’ active and meaningful engagement in physical education learning tasks and includes behavioral, cognitive, and emotional dimensions. From this perspective, PELE is more context-specific and conceptually broader than PAP. At the same time, some overlap may exist between PAP and the behavioral component of PELE, because both involve action- and participation-related expressions. The present findings suggest that PELE should be understood not only in relation to classroom processes, but also in relation to students’ broader behavioral context.

At the same time, digital technology should not be framed in uniformly negative terms. Emerging evidence suggests that well-designed digital health interventions are associated with increases in step counts and improvements in physical activity behavior among university students (Bi et al., 2024). The issue, therefore, may lie less in smartphone use itself than in patterns of use characterized by dependency, loss of control, and excessive reward orientation. A more fine-grained distinction among recreational, academic, and health-promoting smartphone use may help clarify which forms are associated with lower PELE and which may be compatible with, or even supportive of, active lifestyles.

### 4.4. Serial Indirect Association of Self-Esteem and Physical Activity Participation

Beyond the two individual indirect associations, the study also identified a significant serial indirect association involving SE and PAP. Specifically, higher SA was associated with lower SE, lower SE with lower PAP, and lower PAP with lower PELE. Rather than framing SA only as an attentional or time-management issue, this finding points to a broader network of self-related and behavioral correlates.

As a core component of positive self-evaluation, SE is relevant not only to how individuals view themselves but also to their willingness to enter physical activity settings characterized by bodily display, challenge, and social comparison (Ouyang et al., 2019). Individuals with higher SE are generally more confident in their ability to cope with the demands and evaluative pressures of physical activity and are therefore more likely to participate and persist. In contrast, those with lower SE may avoid physical activity because of fear of failure, evaluative concern, or perceived inadequacy (Tang et al., 2025). Previous research has linked SE to adaptive functioning, subjective well-being, persistence, and positive behavior (Li et al., 2025; L. Liu et al., 2024; Neroni et al., 2022; Zheng et al., 2020), while physical activity has been associated with self-control, emotional improvement, and the development of positive lifestyles (Gu et al., 2026; C. Liu, 2020; T. Zhang et al., 2025). From this perspective, the observed serial indirect association linking lower SE, lower PAP, and lower PELE is theoretically coherent.

The findings may also reflect a broader clustering of vulnerability. Greater reliance on smartphones for immediate gratification, challenge avoidance, or external validation may coexist with more fragile self-worth (Nambirajan et al., 2025). Lower SE may in turn be accompanied by reduced confidence and initiative in physical activity, while lower PAP may be associated with fewer opportunities to develop movement competence, experience positive affect, and engage actively in physical education (Hwang & Kim, 2025; Reigal et al., 2024; Tang et al., 2025). However, this pattern should be interpreted as a theoretically coherent set of associations rather than as confirmed evidence of a developmental sequence.

### 4.5. Latent Profile Analysis

The person-centered findings suggest that SA was not entirely homogeneous in this sample. However, the identified profiles appeared to differ mainly in overall severity rather than in qualitatively distinct symptom patterns. Accordingly, the main contribution of the latent profile analysis in the present study may lie in providing a severity-based descriptive framework, rather than in identifying fundamentally different forms of SA. A three-profile solution comprising low-, moderate-, and high-risk groups provided the best fit to the data, and these profiles differed significantly in SE, PAP, and PELE. This suggests that SA may vary meaningfully in severity among university students and that these severity-based subgroups may differ in related psychological and behavioral characteristics.

Whereas the regression analyses estimate average associations under the assumption of population homogeneity, the LPA addresses a different question by testing whether participants can be partitioned into distinct subgroups. In the present sample, the three severity-based profiles provide a risk-stratification framework with quantifiable prevalence estimates (22.10%, 44.24%, and 33.66%) that can inform screening, and the monotonic outcome gradient across profiles indicates that the average associations are not driven by a small atypical subgroup but hold across the severity continuum. The LPA is therefore complementary to, rather than redundant with, the variable-centered findings.

This interpretation is broadly consistent with previous person-centered research. In recent years, several latent class and latent profile studies have documented heterogeneity in problematic smartphone use, with different subgroups showing systematic differences in depression, anxiety, resilience, health-risk behaviors, and psychosocial adjustment (Dong et al., 2025; Gu et al., 2026; Roque Hernández et al., 2025). The present study extends this line of work to the physical education context by showing, from a complementary person-centered perspective, that students in higher-risk SA profiles also reported lower SE, lower PAP, and lower PELE. In this sense, differences in smartphone-addiction severity may coexist with different degrees of vulnerability in physical learning adaptation. Nevertheless, the practical implications of these profiles should be interpreted with caution. Given that they primarily reflect differences in severity rather than qualitatively distinct types of SA, and that their longitudinal stability remains unestablished, these findings offer only limited support for profile-specific or tiered interventions. For now, the profiles are better viewed as a useful descriptive and exploratory framework than as a basis for strong practical recommendations.

## 5. Limitations and Future Prospects

Several limitations should be considered when interpreting the present findings. First, the cross-sectional design precludes firm conclusions regarding temporal ordering and causality. Future studies should use longitudinal, cross-lagged, and intervention-based designs to examine more rigorously the associations among SA, SE, PAP, and PELE. Second, all variables were assessed using a same-source self-report design, which may have increased the risk of common method bias, social desirability effects, and recall error. Although the statistical tests did not suggest that the observed associations were primarily driven by a single common factor, common method bias cannot be entirely excluded. Future research could strengthen the evidence base by employing marker-variable techniques, multi-wave or multi-informant designs, and more diverse data sources, such as objective smartphone-use logs, screen-time records, wearable-device indicators, and teacher ratings. Third, the sample was limited to self-selected respondents from three public universities in a single province of China who were recruited through online platforms. Therefore, the findings should be interpreted cautiously and should not be generalized beyond similar Chinese university contexts. In addition, the online recruitment approach may have introduced self-selection bias, which could affect the representativeness of the sample and the estimated distribution of SA. Future research should replicate these findings across different regions, institutional types, and cultural contexts. Fourth, although this study focused on SE and PAP, the associations examined are likely to be more complex, and reverse causal pathways cannot be excluded. Fifth, information on whether participants were currently enrolled in a PE course was not collected; participants were only required to have current or recent experience with PE classes. Because PE learning engagement is the primary outcome, this limits the validity of its measurement. Future studies should collect current PE enrollment and course characteristics and consider sensitivity analyses restricted to students currently enrolled in PE. Future research may therefore incorporate additional relevant variables to build a more comprehensive explanatory framework. Finally, the long-term stability of the latent profiles identified here remains unclear and should be examined using latent transition analysis or other longitudinal person-centered approaches.

## 6. Conclusions

Using both variable-centered and person-centered approaches, this study examined the associations between SA and PELE among university students. SA was negatively associated with PELE, and SE and PAP were each statistically associated with this relationship, including a significant serial indirect association. The latent profile analysis further identified three severity-based subgroups of SA, which showed significant gradient differences in SE, PAP, and PELE. Together, these findings provide a more nuanced description of how these variables are interrelated. The person-centered results primarily add a severity-based subgroup perspective rather than evidence of qualitatively distinct forms of SA. Accordingly, any implications for differentiated or profile-informed support should be considered preliminary.

## Figures and Tables

**Table 1:** Normality assessment of study variables.

Variables	Skewness	Kurtosis
SA	0.093	−0.450
PELE	0.121	−0.356
SE	0.542	0.488
PAP	0.508	−0.910

Note. SA, smartphone addiction; PELE, physical education learning engagement; SE, self-esteem; PAP, physical activity participation.

**Table 2:** Correlation analysis results.

Variables	Mean	SD	SA	SE	PAP	PELE
SA	40.27	14.24	1			
SE	23.33	5.20	−0.447 **	1		
PAP	40.50	28.56	−0.396 **	0.455 **	1	
PELE	56.64	13.12	−0.353 **	0.405 **	0.415 **	1

** *p* < 0.01.

**Table 3:** Regression analyses among study variables.

Regression Equation		Overall Model Fit			Significance of Regression Coefficients		
Outcome Variable	Predictor Variable	*R*	*R* ^2^	*F*	*β*	*t*	*p*
SE	Gender	0.448	0.201	176.749	0.016	0.839	0.402
	Age				0.006	0.303	0.762
	SA				−0.446	−22.896	<0.001 ***
PAP	Gender	0.532	0.283	208.631	0.164	8.837	<0.001 ***
	Age				0.042	2.295	0.022 *
	SA				−0.231	−11.215	<0.001 ***
	SE				0.344	16.715	<0.001 ***
PELE	Gender	0.503	0.253	129.013	0.053	2.767	0.006 **
	Age				−0.038	−1.989	0.047 *
	SA				−0.152	−7.002	<0.001 ***
	SE				0.223	9.960	<0.001 ***
	PAP				0.244	11.002	<0.001 ***

Note. Gender was coded as 0 = female and 1 = male; *β* = standardized regression coefficient; * *p* < 0.05, ** *p* < 0.01, *** *p* < 0.001.

**Table 4:** Test results of bootstrap indirect effects.

Path	Effect (*β*)	BootSE	BootLLCI	BootULCI	Proportion
Total Effect (SA → PELE)	−0.346	0.019	−0.355	−0.282	—
Direct Effect (SA → PELE)	−0.152	0.020	−0.179	−0.101	—
Total Indirect Effect	−0.194	0.013	−0.221	−0.167	100%
Ind1: SA → SE → PELE	−0.100	0.012	−0.122	−0.076	51.40%
Ind2: SA → PAP → PELE	−0.057	0.007	−0.072	−0.043	29.05%
Ind3: SA → SE → PAP → PELE	−0.038	0.004	−0.046	−0.029	19.55%
Ind1−Ind2	−0.043	0.016	−0.073	−0.012	—
Ind1−Ind3	−0.062	0.013	−0.087	−0.037	—
Ind2−Ind3	−0.019	0.007	−0.033	−0.006	—

Note. Effects are completely standardized coefficients (*β*). Proportions indicate the relative contribution of each indirect effect to the total indirect effect.

**Table 5:** Fit indices for latent profile analysis models.

Model	AIC	BIC	aBIC	LMR (*p*)	BLRT (*p*)	Entropy	Class Proportions%
1-Profile	112,739.882	112,932.262	112,824.240				
2-Profile	99,960.914	100,255.142	100,089.932	<0.0001	<0.0001	0.928	47.31%/52.69%
3-Profile	95,819.804	96,215.880	95,993.482	<0.0001	<0.0001	0.922	22.10%/44.24%/33.66%
4-Profile	93,466.013	93,963.937	93,684.352	<0.0001	<0.0001	0.925	19.83%/35.69%/34.23%/10.25%
5-Profile	92,512.293	93,112.065	92,775.292	0.0518	<0.0001	0.911	19.56%/13.35%/25.72%/31.32%/10.04%

**Table 6:** Distal outcome comparisons across latent profiles using the BCH procedure.

Outcome	C1	C2	C3	Overall *χ*^2^	*p*	C1 vs. C2	C1 vs. C3	C2 vs. C3
SE	27.481 (0.238)	22.672 (0.179)	21.441 (0.141)	478.542	<0.001	251.073 ***	476.382 ***	27.500 ***
PAP	60.688 (1.285)	38.107 (0.965)	30.297 (0.898)	379.793	<0.001	190.160 ***	376.680 ***	33.018 ***
PELE	64.372 (0.605)	55.780 (0.441)	52.665 (0.448)	245.577	<0.001	126.975 ***	242.301 ***	23.108 ***

Note. Values are BCH-estimated means, with standard errors in parentheses. C1, low-smartphone-addiction group; C2, moderate-smartphone-addiction group; C3, high-smartphone-addiction group. *** *p* < 0.001.

**Table 7:** Descriptive effect sizes for pairwise differences in SE, PAP, and PELE across latent profiles.

Outcome	SE	SE	SE	PAP	PAP	PAP	PELE	PELE	PELE
Comparison	C1 vs. C2	C1 vs. C3	C2 vs. C3	C1 vs. C2	C1 vs. C3	C2 vs. C3	C1 vs. C2	C1 vs. C3	C2 vs. C3
Cohen’s *d*	0.90	1.30	0.28	0.79	1.17	0.29	0.65	0.93	0.25

Note. Effect sizes were calculated as Cohen’s *d* based on descriptive statistics from most-likely class assignment.

## Data Availability

The data presented in this study are available from the corresponding author upon reasonable request. The data are not publicly available due to privacy and ethical restrictions involving participant information.

## Supplementary Materials

The following supporting information can be downloaded at https://www.mdpi.com/article/10.3390/bs16091683/s1. Figure S1: Four Latent Profiles of Smartphone Addiction; Figure S2: Five Latent Profiles of Smartphone Addiction.
