# Supplementary material for: Associations Between Smartphone Addiction and Physical Education Learning Engagement Among Chinese University Students: A Variable-Centered and Person-Centered Analysis
Source: Behav Sci (Basel). 2026 Sep 18;16(9):1683. doi: 10.3390/bs16091683 (PMC13603328; doi:10.3390/bs16091683)
Supplement: Supplementary file 1 [file behavsci-16-01683-s001.zip › behavsci-4500346-supplementary.pdf]

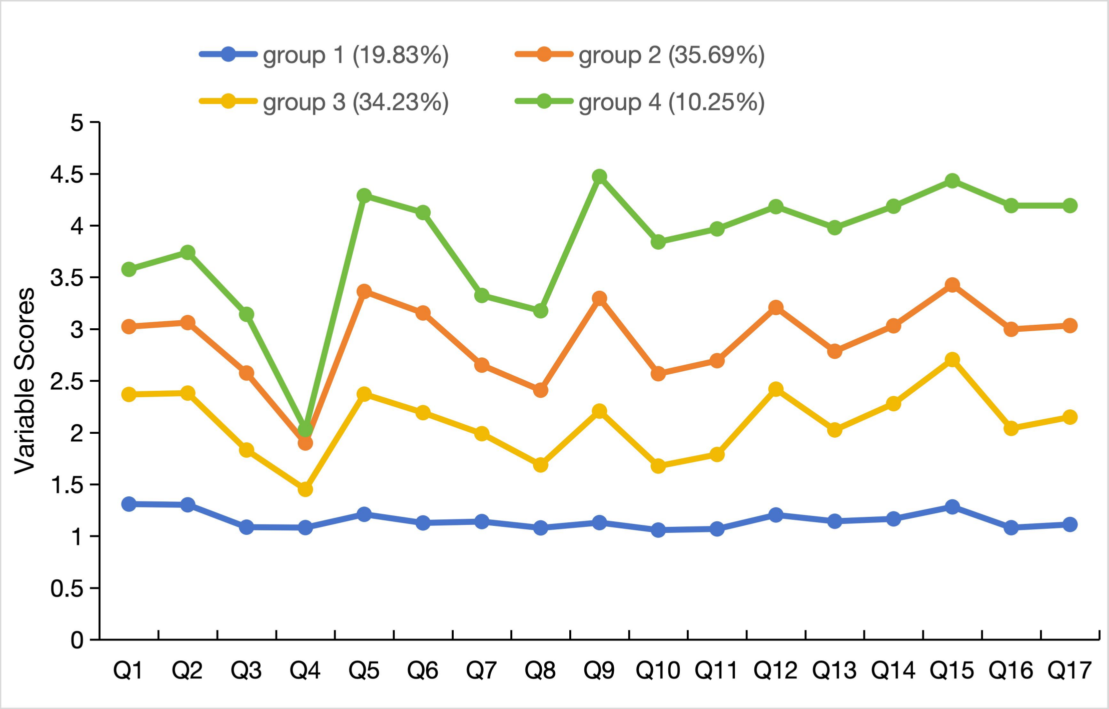

Figure S1. Four Latent Profiles of Smartphone Addiction.

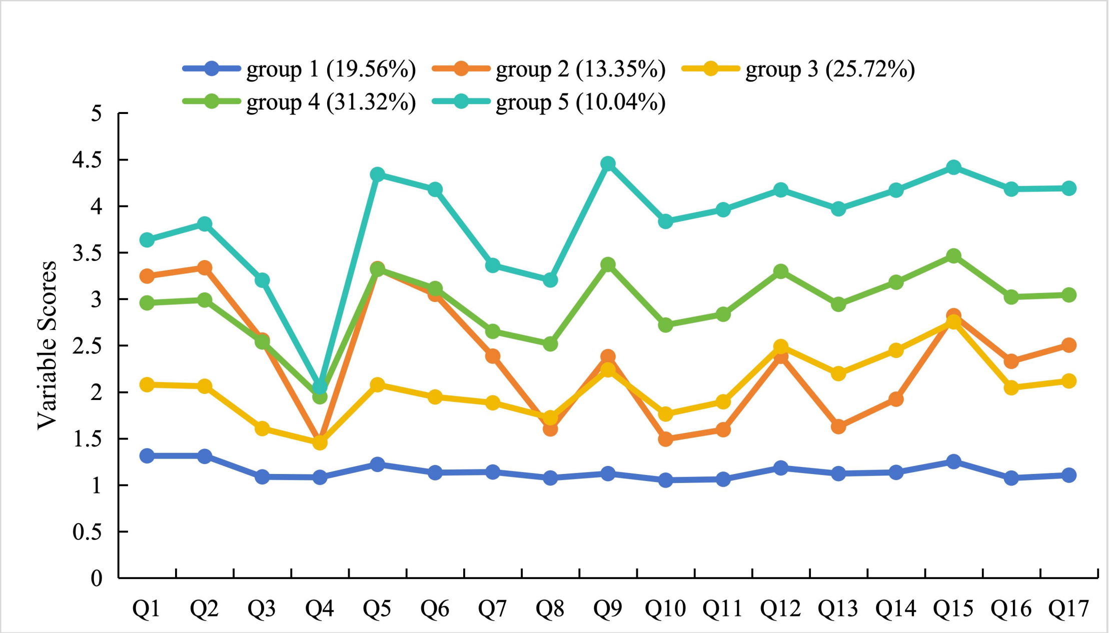

Figure S2. Five Latent Profiles of Smartphone Addiction.
